# CDK4/6 inhibitor-induced bone marrow micronuclei might be caused by cell cycle arrest during erythropoiesis

**DOI:** 10.1186/s41021-024-00298-5

**Published:** 2024-02-01

**Authors:** Yuki Okada, Satsuki Chikura, Takafumi Kimoto, Takeshi Iijima

**Affiliations:** Teijin Institute for Bio-Medical Research, Teijin Pharma Limited, Hino, Tokyo, Japan

**Keywords:** Genotoxicity, In vivo bone marrow micronucleus test, Cell cycle inhibitor, Cyclin-dependent kinase 4/6, False positive

## Abstract

**Background:**

A micronucleus test is generally used to evaluate the genotoxic potential of chemicals. Exaggerated erythropoiesis, as occurs following bleeding, may induce an unexpected increase in micronucleus frequency. This false positive result would be typical in a genotoxicity study due to the enhanced progression of the cell cycle that restores decreased blood cells. The cyclin-dependent kinase (CDK) family is known to play an essential role in preventing genomic instability. Conversely, a selective CDK4/6 inhibitor PD0332991, clinically named Palbociclib, is reported to have genotoxic potential, shown by positive results in both in vitro and in vivo micronucleus studies. To clarify the mechanism by which cell cycle arrest induced by a CDK4/6 inhibitor increases micronucleus frequency, we investigated the positive results of the bone marrow micronucleus test conducted with PD0332991.

**Results:**

Rats treated with PD0332991 exhibited increased micronucleus frequency in an in vivo bone marrow micronucleus test whereas it was not increased by treatment in human lymphoblastoid TK6 cells. In addition, all other genotoxicity tests including the Ames test and the comet assay showed negative results with PD0332991. Interestingly, PD0332991 treatment led to an increase in erythrocyte size in rats and affected the size distribution of erythrocytes, including the micronucleus. The mean corpuscular volume of reticulocytes (MCVr) in the PD0332991 treatment group was significantly increased compared to that of the vehicle control (83.8 fL in the PD0332991, and 71.6 fL in the vehicle control.). Further, the average micronucleated erythrocytes (MNE) size of the PD0332991 group and vehicle control was 8.2 and 7.3 µm, respectively. In the histogram, the vehicle control showed a monomodal distribution with a peak near 7.3 µm. In contrast, the PD0332991 group showed a bimodal distribution with peaks around 7.5 and 8.5 µm. Micronucleated erythrocytes in the PD0332991 group were significantly larger than those in the vehicle control.

These results suggest that the increase in micronucleus frequency induced by the CDK4/6 inhibitor is not due to genotoxicity, but is attributable to disturbance of the cell cycle, differentiation, and enucleation of erythroblasts.

**Conclusions:**

It was suggested that the positive outcome of the in vivo bone marrow micronucleus test resulting from treatment with PD0332991 could not be attributed to its genotoxicity. Further studies to clarify the mechanism of action can contribute to the development of drug candidate compounds lacking intrinsic genotoxic effects.

## Introduction

The in vivo micronucleus test is used to detect chromosome fragments that result from chemical damage to chromosomes and disruptions in chromosome separation caused by the inhibition of spindle formation. Its purpose is to evaluate the presence or absence of genotoxic effects. Currently, the in vivo micronucleus test has been established for various cell types, including cells in the bone marrow (erythroblasts), blood (red blood cells), liver, and gastrointestinal tract [1–4]. Among these, the most commonly used is the in vivo bone marrow micronucleus test, which assesses bone marrow in rodents. This test is employed to identify damage to erythroblast chromosomes and mitotic machinery induced by the test substance [5]. One advantage of using erythroblasts is that micronuclei are easily distinguishable since the main nuclei are enucleated during differentiation into reticulocytes, while the micronuclei remain within the cell [5].

The ICH S2 guidelines caution that the in vivo bone marrow micronucleus test may yield false positive results. Factors that increase division of bone marrow erythroblast cells, such as hypothermia or hyperthermia, glucocorticoids and erythropoietin, can lead to false positives [6, 7]. The precise mechanism by which erythropoietin increases micronucleus frequency is not fully understood. However, studies conducted on mice implanted with erythropoietin-producing tumors and exposed to persistently high levels of erythropoietin have concluded that the increased micronucleus frequency is not due to chromosomal abnormalities, but rather to errors in enucleation and differentiation [8].

Erythrocyte enucleation occurs when the erythroblast cell cycle is arrested [9]. Erythropoietin is known to increase the levels of p27kip1, which in turn arrests the cell cycle by inhibiting CDK [10]. Therefore, it is possible that inhibition of targets involved in the cell cycle, such as CDKs, and induction of cell cycle arrest may lead to errors in enucleation and an increase in micronucleus frequency.

Since kinases are involved in cell cycle progression and chromosomal dynamics, inhibition of kinases can lead to cell cycle arrest, abnormal progression, and abnormal chromosome segregation [11]. Specifically, compounds that inhibit cyclin-dependent kinases (CDKs) 2, 3, 5 are known to increase micronucleus frequency [12, 13]. CDK, a family of CMGC (Acronym for a group of kinases including CDK, MAPK, GSK3, and CLK) kinases associated with mitosis and the cell cycle, tightly regulates the cell cycle by binding to cyclin at various stages of cell division [14, 15]. For example, CDK1 forms a complex with cyclin A and is essential for transitioning from G2 to M [16]. CDK2 similarly forms a complex with cyclin A and plays an important role in S phase progression [17, 18]. CDK4 and CDK6 form complexes with cyclin D and initiate phosphorylation of the retinoblastoma protein family, leading to E2F release and cell cycle progression [19–22].

Therefore, it is suggested that deviation from cell cycle control and micronuclei formation are closely related. PD0332991, a selective CDK4/6 inhibitor, induces arrest of the cell cycle and has been reported to show genotoxic potential resulting in positive results in both in vitro and in vivo micronucleus studies [23]. We therefore investigated the mechanism by which cell cycle arrest by CDK4/6 inhibition leads to micronucleus induction.

## Materials and methods

### Test compounds and animal experiments

PD0332991 (Lot No:6HGC701, 6X35101, purity: > 99.12%) was purchased from Shanghai Sun-shine Chemical Technology Corporation Limited (Wuhan, China). Dimethyl sulfoxide and 0.5% methylcellulose were used as solvents for in vitro studies and in vivo studies, respectively.

Specific pathogen-free Male Crl: CD (SD) rats were purchased from CHARLES RIVER LABORATORIES JAPAN, INC. (Kanagawa, Japan). The rats were housed in a controlled environment with a 12-h light/dark cycle and standard food and water were provided ad libitum. All animal experiments were approved by the Animal Care and Use Committee of Teijin Pharma Limited.

### Genotoxicity studies

All genotoxicity studies were conducted in accordance with the principles of the Organization for Economic Co-operation and Development (OECD) test guidelines (TG).

The Ames test was conducted according to OECD TG471. Briefly, TA98, TA100, and WP2*uvrA* bacterial strains were treated using the preincubation method in the presence and absence of a metabolic activation system.

The in vitro micronucleus test was conducted according to OCED TG487 [24]. Briefly, TK6 cells were cultured and subjected to short-term and continuous treatment without a metabolic activation system. In the short-term treatment, the cells were cultured for 3 h with PD0332991. PD0332991 was then removed, and the cells were cultured for 21 h for recovery. In the continuous treatment, the cells were cultured for 24 h with PD0332991. In both cases, cytochalasin B was added 21 h before sample preparation in the short-term treatment and 24 h before sample preparation in the continuous treatment. After culture, specimens were prepared and examined with acridine orange (AO) staining. A total of 500 cells were counted to determine cytotoxicity and the cytokinesis-block proliferation index (CBPI) was calculated. A total of 1,000 binucleated cells were counted to determine the frequency of micronuclei.

The in vivo micronucleus and comet assays were conducted according to OECD TG474, TG489, and the International Validation Trial Protocol [5, 25, 26]. Briefly, eight-week-old male rats (*n* = 6/group) were treated with PD0332991 or vehicle control three times at 24 h intervals. Cyclophosphamide and ethyl methanesulfonate were used as positive controls. Specimens were prepared from bone marrow and liver 4 h after administration of the final dose, except for cyclophosphamide, where specimens were collected 24 h post-administration. Bone marrow cells were fixed with formalin, stained with AO, and examined under a microscope. Livers were sampled according to the International Validation Test Protocol [25], stained with SYBR Gold, and then cells were counted using the Comet Assay IV (Instem, Staffordshire, UK). For the in vivo bone marrow micronucleus test, 1000 erythrocytes were counted to determine the ratio of polychromatic erythrocytes (PCE) to total erythrocytes, and 2000 PCE were counted to determine the ratio of micronucleated PCE (MNPCE) to total PCE. Among MNPCE, micronuclei smaller than 1/4 of the cell diameter were classified as small (PS), and those between 1/4 and 1/2 of the cell diameter as large (PL). In the comet assay, 200 electrophoretic images were measured to determine the percentage of DNA in the tail.

The in vivo chromosome aberration test was conducted under conditions in which PD0332991 previously showed positive results in the in vivo bone marrow micronucleus test, according to OECD TG475 [27]. Briefly, eight-week-old male rats (*n* = 6/group) were treated with PD0332991 or vehicle control twice every 24 h. Cyclophosphamide was used as a positive control. Bone marrow cells were collected 24 h after the final administration and specimens were prepared. A single intraperitoneal administration of colchicine was performed 2–3 h before collection of bone marrow cells. For bone marrow, nucleated cells were counted using ADVIA120 (Siemens Healthcare Diagnostics K.K., Tokyo, Japan). For structural aberration and polyploidy analyses, specimens were stained with Giemsa and examined under a microscope. A total of 200 metaphase cells were counted to determine the frequency of chromosomal aberrations and polyploidy.

### Genotoxicity studies or statistical analysis

Six-week-old male rats (*n* = 5 /group) were administered PD0332991 or vehicle control three times every 24 h. Blood was collected 24 h after the final administration, and the MCVr was measured using ADVIA120. Ten MNE per individual were counted using bone marrow smear slides to determine the size of MNE. The size was defined as the average of the minor axis and the major axis.

### Statistical analysis

SAS / EXSUS (CAC Croit Corporation, Tokyo, Japan) was used for statistical analyses. The %MNPCE in the in vivo bone marrow micronucleus test was analyzed by Fisher’s exact test and Bonferroni’s correction and compared to the vehicle control (significance level: 2.5% on one side). The %PCE, and the number of PS and PL were compared between the vehicle control and the PD0332991-treated groups and analyzed by Dunnett’s test. The difference in the number of PS and PL between the vehicle control and positive control group was analyzed by Student’s t test. (significance level: 5%).

The percentage of DNA in the tail in the vehicle control compared to the PD0332991 groups was analyzed by Dunnett’s test (significance level: 5%).

The micronucleus frequency in the in vitro micronucleus test was analyzed by Fisher’s exact test and Bonferroni’s correction (significance level: 2.5% on one side).

Chromosomal aberration frequency and polyploidy frequency in the in vivo chromosome aberration test were analyzed by Fisher’s exact test (significance level: 5% on one side). The number of bone marrow nucleated cells in the vehicle control compared to each PD0332991 treatment group was analyzed by Dunnett’s test (significance level: 5%).

MCVr and the size of MNE were analyzed by Student’s t-test (significance level: 5%).

## Results

### In vivo bone marrow micronucleus test (Fig. 1)

**Fig. 1:**
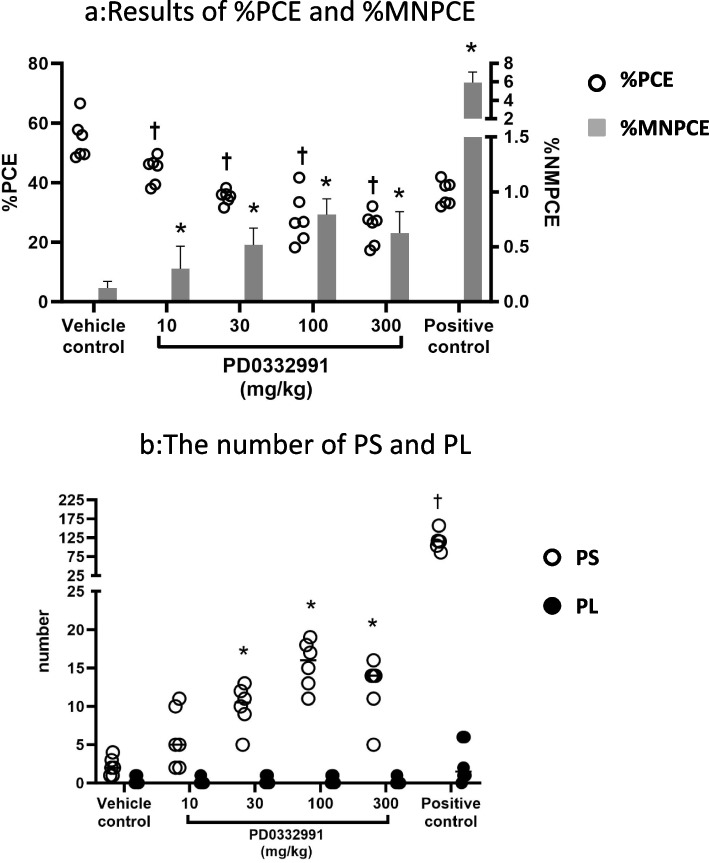
Results of the in vivo bone marrow micronucleus test for the selective CDK4/6 inhibitor PD0332991 using rats. **a** Results of the %PCE and %MNPCE. White circles indicate individual %PCE, gray bars indicate mean of %MNPCE and error bars indicate standard deviation. † and * indicate statistically significant differences (†: Dunnett’s test compared with the vehicle control. Significance level was 5%, *: Fisher’s exact test and Bonferroni’s correction compared with the vehicle control. Significance level was 2.5% one-sided). **b** The number of PS and PL. White circles indicate individual PS, black circles indicate individual PL. * and † indicate statistically significant differences (*: Dunnett’s test compared with the vehicle control. †: Student’s t-test compared with the vehicle control. Significance level was 5%)

The %PCE was significantly decreased in all PD0332991-treated groups compared with the vehicle control. The 300 mg/kg group, which exhibited the greatest decrease, had an approximately 50% reduction in %PCE in comparison to the vehicle control. The %MNPCE in the vehicle control, PD0332991 groups at 10, 30, 100, and 300 mg/kg, and the positive control was 0.13, 0.30, 0.52, 0.79, 0.63 and 5.92%, respectively (Fig. 1a). Consequently, treatment with PD0332991 led to a significant increase in %MNPCE in all groups, compared to the vehicle control. The 100 mg/kg group, which exhibited the greatest increase in %MNPCE, had approximately 6X higher %MNPCE compared to the vehicle control. Thus, PD0332991 was shown to induce micronuclei in vivo.

The number of PS in the vehicle control, PD0332991-treated groups at 10, 30, 100, and 300 mg/kg, and the positive control was 0.11, 0.29, 0.50, 0.78, 0.62 and 5.79%, respectively. The increase in PS number was significant in the 30, 100, and 300 mg/kg PD0332991 treatment groups compared with the vehicle control. There were, however, no differences in the number of PL between the vehicle control and PD0332991 groups (Fig. 1b).

### Other genetic toxicity tests

#### Ames test (Fig. 2a)

**Fig. 2:**
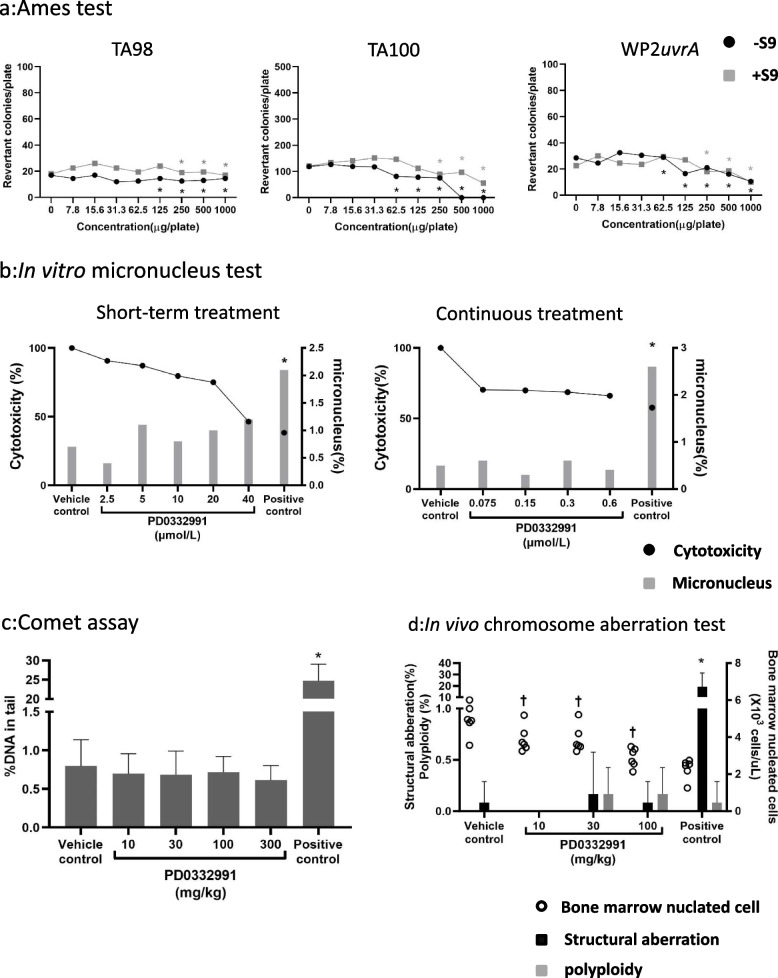
Results of genotoxicity tests for the selective CDK4/6 inhibitor PD0332991. **a** Ames test. Each dot indicates the mean of 2 plates and * indicates growth inhibition. **b** In vitro micronucleus test. Black dots indicate cytotoxicity, expressed as CBPI relative to the negative control (100%). Gray bars indicate the micronucleus frequency. * indicates a statistically significant difference (*: Fisher’s exact test and Bonferroni’s correction. Significance level was 2.5% one-sided). **c** Comet assay. Gray bars indicate the mean %DNA in tail and error bars indicate standard deviation. * indicates a statistically significant difference (*: Dunnett’s test compared with the vehicle control. Significance level was 5%). **d** In vivo chromosome aberration test. White circles indicate the individual number of bone marrow nucleated cells. Black bars indicate the mean structural aberration frequency and error bars indicate standard deviation. Gray bars indicate the mean polyploidy frequency and error bars indicate standard deviation. † and * indicate statistically significant differences (†: Dunnett’s test compared with the vehicle control. Significance level was 5%, *: Fisher’s exact test and Bonferroni’s correction compared with the vehicle control. Significance level was 2.5% one-sided)

In the Ames test, growth inhibition was observed at 250 µg/plate or higher in all strains and series and the increase in the number of revertant colonies did not exceed twofold that of the vehicle control in all strains and series. Based on this finding, PD0332991 was determined to be negative in this assay.

#### In vitro micronucleus test (Fig. 2b)

##### Short-term treatment

Cytotoxicity of the vehicle control, PD0332991 groups at concentrations of 2.5, 5, 10, 20, and 40 µmol/L, and the positive control was 100, 90.7, 87.2, 79.6, 75.1, 46.4, and 38.3%, respectively. The micronucleus frequency of the vehicle control, PD0332991 groups at 2.5, 5, 10, 20, and 40 µmol/L and the positive control was 0.7, 0.4, 1.1, 0.8, 1.0, 1.2, and 2.1%, respectively. Notably, in all PD0332991 groups, there was no significant increase in micronucleus frequency compared to the vehicle control.

##### Continuous treatment

Cytotoxicity of the vehicle control, PD0332991 groups at concentrations of 0.075, 0.15, 0.3, and 0.6 µmol/L, and the positive control was 100, 70.3, 69.9, 68.6, 66.1, and 57.6%, respectively. The micronucleus frequency of the vehicle control, PD0332991 groups at 0.075, 0.15, 0.3, and 0.6 µmol/L, and the positive control was 0.5, 0.6, 0.3, 0.6, 0.4, and 2.6%, respectively. In all PD0332991 groups, there was no significant increase in micronucleus frequency compared to the vehicle control. Based on these results, PD0332991 was determined to be negative in the in vitro micronucleus test.

#### Comet assay (Fig. 2c)

In the Comet assay the percentage of DNA in the tail of the vehicle control, PD0332991 groups at 10, 30, 100, and 300 mg/kg, and the positive control was 0.80, 0.70, 0.69, 0.72, 0.61, and 24.72%, respectively. There was no significant increase in percentage of DNA in the tail compared to the vehicle control at the same dose used in the in vivo bone marrow micronucleus test. Consequently, PD0332991 was judged to be negative in the Comet assay.

#### In vivo chromosome aberration test (Fig. 2d)

In the chromosome aberration test, the number of nucleated bone marrow cells was significantly decreased in all PD0332991 groups compared with the vehicle control. The frequency of structural aberration of the vehicle control, PD0332991 groups at 10, 30, and 100 mg/kg, and the positive control was 0.08, 0, 0.17, 0.08, and 19.1%, respectively. The polyploidy frequency of the vehicle control, PD0332991 groups at 10, 30, and 100 mg/kg, and the positive control was 0, 0, 0.17, 0.17 and 0.08%, respectively. There was a significant decrease in bone marrow nucleated cells at all doses. For all PD0332991 groups, there was no significant increase in either structural aberrations or polyploidy compared to the vehicle control. Based on these results, PD0332991 was determined to be negative.

### MCVr (Fig. 3)

**Fig. 3:**
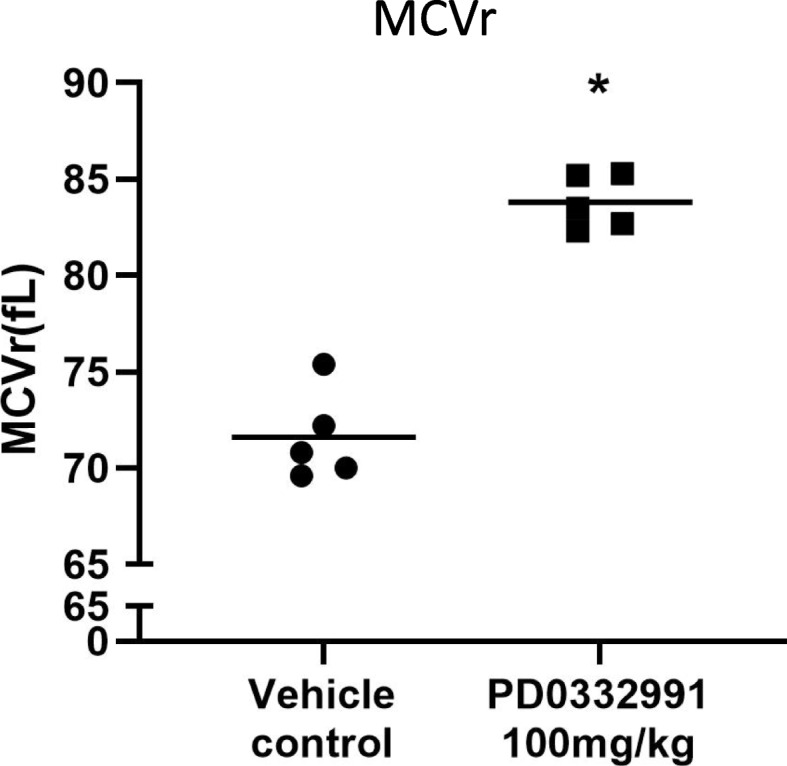
Results of short-term (3-day) PD0332991 treatment on MCVr. Black circles indicate individual values for MCVr in the vehicle control and black bars indicate individual values for MCVr in the PD0332991 treatment group. * indicates a statistically significant difference (*: Student’s t-test. Significance level was 5%)

The MCVr was significantly increased in the PD0332991 group, which had a value of 83.8 fL compared to the vehicle control, with a value of 71.6 fL.

### MNE size (Fig. 4)

**Fig. 4:**
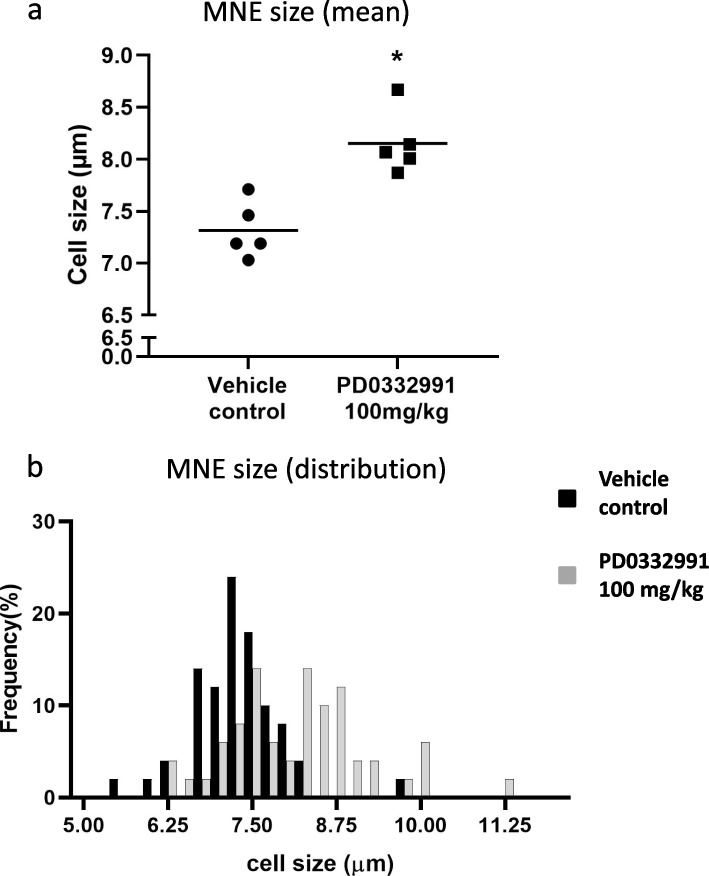
Results of short-term (3-day) PD0332991 treatment on MNRBC size. **a** Black circles indicate individual values for MNRBC in the vehicle control and black squares indicate individual values for MNRBC in the PD0332991 treatment group. * indicates a statistically significant difference (*: Student’s t-test. Significance level was 5%). **b** MNRBC distribution. Black bars and gray bars show the ratio of all MNRBC included in the vehicle control and PD0332991 treatment groups, respectively

The average MNE size in the PD0332991 group and the vehicle control was 8.2 and 7.3 µm, respectively. In the histogram, the vehicle control exhibited a monomodal distribution with a peak near 7.3 µm. In contrast, the PD0332991 group had a bimodal distribution with peaks around 7.5 and 8.5 µm. Micronucleated erythrocytes in the PD0332991 group were significantly larger than those in the vehicle control.

## Discussion

The genotoxicity test results for PD0332991 were negative in the Ames test, negative in the in vitro chromosome aberration test using human cells, positive in the in vitro micronucleus test using CHO-WBL cells, and positive in the in vivo bone marrow micronucleus test [23]. The in vitro micronucleus test using CHO-WBL cells showed an increase in centromere-positive micronuclei, which led to the conclusion that aneuploidy was the cause of the positive outcome in the in vivo micronucleus test. The treatment concentration for the in vitro micronucleus test using CHO-WBL cells was 35 µg/mL for the 24 h continuous treatment, while in the in vitro micronucleus test using human cells, the concentrations ranged from 0.331 to 1.57 µg/mL for continuous treatment over 22 h. The minimum C_max,total_ and C_max,u_ at which the in vivo micronucleus test yielded a positive result, was approximately 2220 ng/mL and 277.5 ng/mL using a dose of 100 mg/kg/day, respectively.

Although PD0332991 is a selective CDK4/6 inhibitor, it inhibits several kinases including FLT3, DYRK1B, and CAMK2D with greater than 50% inhibition when used at a dose of approximately 450 ng/mL. The treatment concentration used in the in vitro micronucleus test using CHO-WBL cells far exceeded this concentration. It has been reported that the results of in *vitro* micronucleus tests can be predicted with 85% accuracy using machine learning methods, based on the inhibition profiles of 21 kinases, [28]. Notably, FLT3, DYRK1B, and CAMK2D were among those 21 kinases.

Based on information above, it is possible that the increased micronuclei observed in the in vitro micronuclei test using CHO-WBL cells was not due to CDK4/6 inhibition, but was a result of off-target effects due to inhibition of other kinases. In this case, these results cannot be extrapolated to the in vivo micronuclei test results.

To investigate the effects of CDK4/6 inhibition on chromosomes, an in vivo bone marrow micronucleus test was performed in rats using PD0332991. The in vivo bone marrow micronucleus test is widely used to examine the effect of compounds on chromosomes. PD0332991 is considered to have chromosomal toxicity when the frequency of red blood cells (reticulocytes) containing micronuclei increases [5, 29]. PD0332991 significantly increased the frequency of reticulocytes with micronuclei. Structural abnormalities or aneuploidy were considered to be the mechanism underlying the increase in micronucleus frequency. Centromere staining with a molecular probe or fluorescent labeled antibody, can be used to distinguish whether a micronuclei is derived from structural aberration or aneuploidy [30]. However, we were unable to perform this staining because a rat pan-centromere probe was not commercially available and the maximum micronucleus frequency was only 0.79%, which did not allow for sufficient staining.

An alternative method for determining structural abnormalities and aneuploidy is to compare the frequency of large micronuclei with that of small micronuclei [31]. Our investigation of the specimens in the rat micronucleus study revealed an increase in the number of PS, but not PL. Therefore, aneuploidy is unlikely to be the mechanism for the increase in micronucleus frequency observed in the in vivo micronucleus test.

To investigate whether the increase in micronucleus frequency is due to structural abnormalities, the Ames test, comet assay using rat liver, and in vivo bone marrow chromosomal abnormality test were conducted. Since PD0332991 was negative in all of these tests, it was concluded that PD0332991 did not induce structural abnormalities. Subsequently, an in vitro micronucleus test using TK6 cells was performed to investigate aneuploidy induction. Since it is known that there are no chemical substances that induce aneuploidy by only metabolic activation [32], short-term and continuous treatments without metabolic activation were performed, and the results were negative. The ratio of large to small micronuclei in the in vivo bone marrow micronucleus test was similar to the ratio of those that occur spontaneously (data not shown), and the frequency of polyploidy did not increase in the in vivo chromosomal aberration test. Based on these results, the likelihood of aneuploidy induction was considered to be low.

From the results above, it was hypothesized that the micronucleus frequency might have increased due to a mechanism other than genotoxicity.

In the in vivo bone marrow micronucleus test, false positives are known to occur as a result of errors in differentiation and enucleation caused by fluctuations in body temperature and increased hematopoiesis [33]. Mammalian erythrocytes differentiate from hematopoietic stem cells into erythrocytes via BFU-E and CFU-E [34–36]. In the final stage of differentiation, the size of erythroblasts gradually decreases during repeated cell divisions, and finally, the nucleus concentrates and enucleates during arrest of the cell cycle [19, 34–36]. CDK4/6 is an essential factor in advancing the cell cycle from the G1 to S phase [37]. Cell cycle arrest induced by CDK4/6 inhibitor leads concentration of the nucleus and enucleation. Previous studies have indicated that the presence of micronuclei results in apoptotic cell death [38, 39]. Therefore, erythroblasts with micronuclei produced during the proliferative stage are anticipated to be removed during the next cell division. In the in vivo bone marrow micronucleus test, erythrocytes with micronuclei formed during the last division and the main nucleus enucleated are counted as MNE. CDK4/6 inhibition is thought to lead to cell cycle arrest, which may lead to nuclear concentration and enucleation even during the differentiation stage, where nuclear concentration and enucleation do not normally occur initially. Therefore, we hypothesized that spontaneous erythroblasts with micronuclei, which are initially eliminated via apoptosis, would also differentiate into erythrocytes with micronuclei via early enucleation, potentially elevating the frequency of erythrocytes with micronuclei.

Cyclin is an essential factor that forms a complex with CDK to advance the cell cycle. Multiple cyclin subtypes such as cyclin A, D, and E are known [40]. An increase in MCV in peripheral blood was observed in cyclin A2 and cyclin D3 knockout mice. Moreover, an increased frequency of peripheral blood micronuclei has also been observed in cyclin A2 knockout mice [41, 42]. These observations indicate that cyclin A2 and cyclin D3 are essential for cell division, and their deficiency causes cell cycle arrest. The increased frequency of peripheral blood micronuclei observed in cyclin A2 knockout mice causes abnormal differentiation and enucleation of erythroblasts by arresting the normal cell cycle during erythropoiesis. This information appears to support a possible mechanism for the observed increase in micronucleus frequency observed with PD0332991 in this study. Therefore, we investigated the effects of PD0332991 on erythrocyte differentiation and enucleation, a result similar to the increased peripheral blood MCV observed in cyclin A2 and cyclin D3 knockout mice.

To examine abnormalities in differentiation, peripheral blood was analyzed at the dose and duration at which increased micronuclei were observed. The results showed an increase in reticulocyte MCV, although whole red blood cell MCV did not increase because of the short dosing period (3 days). The lifespan of erythrocytes in rats is reported to be approximately 60 days [43, 44] and the number of erythrocytes in the circulating blood is far greater than the number supplied during the three-day administration period. Supporting evidence for this comes from the observation of an increase in peripheral blood MCV with PD0332991 following 2 weeks of dosing (data not shown). Thus, it is likely that the increase in peripheral blood MCV observed with PD0332991 was due to increased enucleation during a state of inhibited cell division, similar to that observed in cyclin A2 knockout erythrocytes [45].

To analyze the relationship between increased micronucleus frequency induced by PD0332991 and increased MCV, the size of micronucleated erythrocytes induced by administration of PD0332991 was examined. The results showed that the size of erythrocytes with micronuclei that occurred spontaneously was 7.3 µm, whereas the size of erythrocytes with micronuclei induced by PD0332991 was significantly larger at 8.1 µm. In addition, the size of micronucleated erythrocytes induced by PD0332991 was bimodal. One peak showed a distribution similar to that seen with spontaneous micronucleation, whereas the other peak was more extensive than that observed with spontaneous micronucleation. The first peak was thought to represent spontaneous micronuclei, while the second peak was considered to be the result of enucleation before reaching the size typical for enucleation. Consequently, it was concluded that the increase in micronucleus frequency by PD0332991 was due to the progression of enucleation during a state of inhibited division, similar to the observed increase in MCV.

Terminal differentiation of erythroblasts refers to the period from the proliferation of erythroblasts in CFU-E to enucleation. During terminal differentiation, erythroblasts divide about 3–4 times, then stop dividing and undergo nuclear concentration and enucleation [46]. Nuclear concentration is a gradual change in erythroblasts that is known to be promoted by histone deacetylases (HDACs) and inhibited by Gcn5 [34]. The expression level of Gcn5 is also known to be significantly reduced in the late stage of erythroblast differentiation [47]. Furthermore, it has been shown that cyclin D1-CDK4 directly phosphorylates and activates Gcn5 in vitro, and a CDK4 inhibitor inhibits Gcn5 phosphorylation [48].

The presumed events leading to PD0332991-induced increase in micronucleus frequency is as follows: PD0332991 causes G1 arrest by inhibiting CDK4/6 in erythroblasts. It would also lead to reduced expression of Gcn5. In erythroblasts with cell cycle arrest and reduced Gcn5 expression, concentration of chromatin initiates at an earlier stage, and nuclear concentration and enucleation proceed. The following are possible causes for the presence of CDK4/6 inhibitor treatment-induced micronuclei:

- 1. Chromosomes may be lost during the enucleation process due to enucleation with incomplete chromosome concentration.
- 2. Not all nuclei can be enucleated because they are in a state where their nuclei are larger than those that are typically enucleated.
- 3. Erythroblasts with spontaneous micronuclei are usually removed by apoptosis because chromosomal integrity is not maintained. However, with PD0332991, these cells do not undergo apoptosis but are enucleated instead, becoming red blood cells that contain micronuclei.

**Fig. 5:**
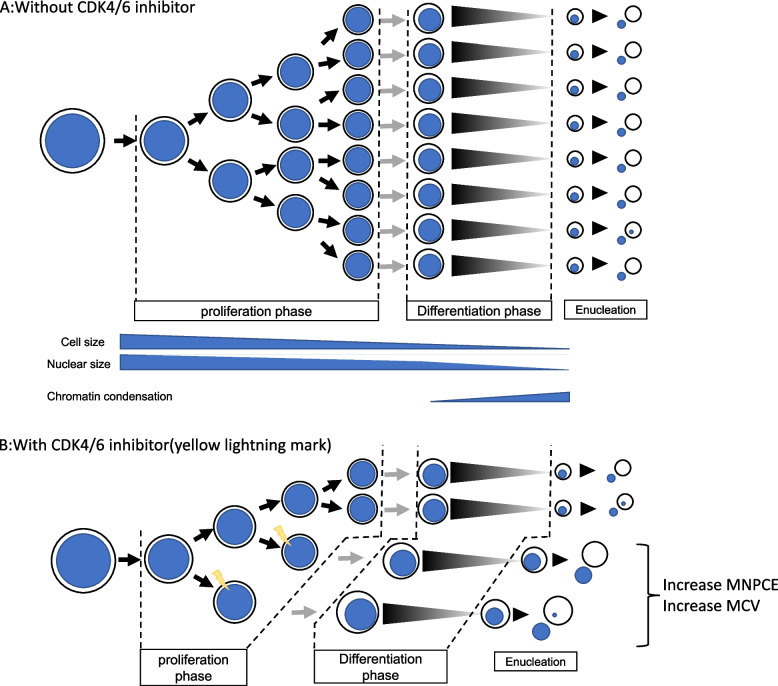
Hypothesized mechanism by which CDK4/6 inhibitors increase micronucleus frequency. **A **Without CDK4/6 inhibitor. The differentiation of erythrocytes is divided into three phases. The first is the proliferation phase, in which cells divide to form hematopoietic stem cells, proerythroblasts, basophilic erythroblasts, polychromatic erythroblasts, and orthochromatic erythroblasts. The second phase is the differentiation phase, in which no further cell division occurs, and chromatin condensation begins. The third phase is the enucleation phase, in which the chromatin is sufficiently condensed and enucleation occurs to form reticulocytes. In the proliferation phase, cell size and nucleus size decrease with division. In the differentiation phase, nucleus size decreases rapidly due to chromatin condensation. Micronuclei occur spontaneously at a certain rate during mitosis, and erythroblasts with micronuclei are removed by apoptosis by the time of the next mitosis. Only the main nucleus is enucleated and micronuclei is not enucleated. **B** With CDK4/6 inhibitor. Cells in which CDK4/6 is inhibited (yellow lightning mark) stop the cell cycle, exit the proliferation phase, and enter the differentiation phase. Cells enucleate before the cell size decreases and before chromatin becomes sufficiently small and condensed. Therefore, micronuclei are likely to be left in the cell, thus increasing the likelihood of micronuclei forming. In addition, MCV increases because the cells undergo enucleation before they reach a sufficiently small size

These mechanisms taken together suggest that the increase in micronucleus frequency by the CDK4/6 inhibitor is not due to genotoxicity, but is attributable to disturbance of the cell cycle, differentiation, and abnormal enucleation of erythroblasts. The MoA was thought to be due to the pharmacological effect of PD0332991, which leads to G1 arrest. Figure 5 shows the putative mechanism for the increase in MNRBC by PD0332991. The differentiation of erythrocytes is divided into three phases. The first is the proliferation phase, in which cells divide to form hematopoietic stem cells, proerythroblasts, basophilic erythroblasts, polychromatic erythroblasts, and orthochromatic erythroblasts. The second phase is the differentiation phase, in which cell division stops and chromatin condensation begins. The third phase is the enucleation phase, in which the chromatin is sufficiently condensed and enucleation occurs to form reticulocytes. In the proliferation phase, cell size and nucleus size decrease with division. In the differentiation phase, nucleus size decreases rapidly due to chromatin condensation. Cells in which CDK4/6 is inhibited exhibit cell cycle arrest and exit the proliferation phase and enter the differentiation phase. In this case, cells enucleate before the cell size is adequately decreased and chromatin is sufficiently condensed. Therefore, micronuclei are likely to be left in the cell which increases the number of micronuclei formed. In addition, MCV increases because the cells are enucleated before they decrease in size.

The results suggest that the number of cells with micronuclei not attributable to chromosomal damage might have increased. To evaluate this hypothesis, future research will investigate which stage of differentiation is affected by CDK4/6 inhibitor-induced cell cycle arrest and will determine chromatin enrichment in erythroblasts upon cell cycle arrest. Moreover, investigation of CDK4/6 inhibitor-induced alterations in the expression of enzymes involved in chromatin regulation (Gcn5 and HDAC) is necessary to confirm this hypothesis.

This study revealed that erythroblast cell cycle disruption can lead to positive results in micronucleus tests, which are not due to genotoxicity. We suggest that clarifying the mechanism of action (MoA) through studies such as the present one, can contribute to the development of drug candidate compounds that do not have intrinsic genotoxic effects.

## Conclusion

Under the conditions tested, we observed a statistically significant increase in micronucleus frequency in the rat bone marrow treated with PD0332991. We clarified the possibility that this increase resulted from inhibition of erythrocyte differentiation and proliferation caused by cell cycle arrest based on CDK4/6 inhibition, which is a pharmacological action of PD0332991. The present study demonstrated that the cell cycle arrest in erythropoiesis could affect in vivo MN test results. The finding provides one of the helpful information to interpret positive results of in vivo MN test in drug development.

## Data Availability

The data that support the findings of this study are available from the corresponding author, TK, upon reasonable request.

## Abbreviations

AO: Acridine orange
CBPI: Cytokinesis-block proliferation index
CDK: Cyclin dependent-kinase
HDAC: Histone deacetylase
MCV: Mean corpuscular volume
MCVr: Mean corpuscular volume of reticulocyte
MNPCE: Micronucleated polychromatic erythrocyte
MNE: Micronucleated erythrocyte
MoA: Mechanism of action
OECD: Organization for Economic Co-operation and Development
PCE: Polychromatic erythrocyte
PL: Polychromatic erythrocyte contained large micronuclei
PS: Polychromatic erythocyte contained small micronuclei
RBC: Red blood cell
TG: Test guideline

## References

[1] Jain AK (2019). In vivo micronucleus assay in mouse bone marrow. Methods Mol Biol.

[2] Kasahara Y (1992). The micronucleus test using peripheral blood reticulocytes from methotrexate-treated mice. Mutat Res.

[3] Hamada S (2015). Evaluation of the repeated-dose liver and gastrointestinal tract micronucleus assays with 22 chemicals using young adult rats: summary of the collaborative study by the Collaborative Study Group for the Micronucleus Test (CSGMT)/The Japanese Environmental Mutagen Society (JEMS) - Mammalian Mutagenicity Study Group (MMS). Mutat Res Genet Toxicol Environ Mutagen.

[4] Okada E (2008). Detection of micronucleated cells and gene expression changes in glandular stomach of mice treated with stomach-targeted carcinogens. Mutat Res.

[5] OECD. Test No.474: Mammalian Erythrocyte Micronucleus Test: OECD Guidelines for the Testing of Chemicals. Paris: OECD; 2016. 10.1787/9789264264762-en.

[6] Tweats DJ (2007). Report of the IWGT working group on strategies and interpretation of regulatory in vivo tests I Increases in micronucleated bone marrow cells in rodents that do not indicate genotoxic hazards. Mutat Res.

[7] Julie E (2013). Micronucleus induction in the bone marrow of rats by pharmacological mechanisms. I: glucocorticoid receptor agonism. Mutagenesis.

[8] Yajima N (1993). Genotoxicity of genetic recombinant human erythropoietin in a novel test system. Mutagenesis.

[9] Hsieh FF (2000). Cell cycle exit during terminal erythroid differentiation is associated with accumulation of p27(Kip1) and inactivation of cdk2 kinase. Blood.

[10] Sherr C J (1999). CDK inhibitors: positive and negative regulators of G1-phase progression. Genes Dev.

[11] Vieth M (2005). Kinomics: characterizing the therapeutically validated kinase space. Drug Discovery Today.

[12] Olaharski AJ (2009). Identification of a Kinase Profile that Predicts Chromosome Damage Induced by Small Molecule Kinase Inhibitors. PLoS Comput Biol.

[13] Hughes BT (2013). Essential role for Cdk2 inhibitory phosphorylation during replication stress revealed by a human Cdk2 knockin mutation. Proc Natl Acad Sci U S A.

[14] Malumbres M (2014). Cyclin-dependent kinases. Genome Biol.

[15] Satyanarayana A, Kaldis P (2009). Mammalian cell-cycle regulation: several Cdks, numerous cyclins and diverse compensatory mechanisms. Oncogene.

[16] Furuno N, den Elzen N, Pines J (1999). Human cyclin A is required for mitosis until mid prophase. J Cell Biol.

[17] Petersen BO (1999). Phosphorylation of mammalian CDC6 by cyclin A/CDK2 regulates its subcellular localization. EMBO J.

[18] Oehlmann M, Score AJ, Blow JJ (2004). The role of Cdc6 in ensuring complete genome licensing and S phase checkpoint activation. J Cell Biol.

[19] Sherr CJ, Roberts JM (1999). CDK inhibitors: positive and negative regulators of G1-phase progression. Genes Dev.

[20] Sherr CJ, Roberts JM (2004). Living with or without cyclins and cyclin-dependent kinases. Genes Dev.

[21] Weinberg RA (1995). The retinoblastoma protein and cell cycle control. Cell.

[22] Dyson N (1998). The regulation of E2F by pRB-family proteins. Genes Dev.

[23] Ibrance (palbociclib) Capsules, Drug Approvals and Databases. https://www.accessdata.fda.gov/drugsatfda_docs/nda/2015/207103Orig1s000PharmR.pdf.

[24] OECD. Test No.487: In Vitro Mammalian Cell Micronucleus Test: OECD Guidelines for the Testing of Chemicals. Paris: OECD; 2016. 10.1787/9789264264861-en.

[25] Uno Y (2015). JaCVAM-organized international validation study of the in vivo rodent alkaline comet assay for the detection of genotoxic carcinogens: I. Summary of pre-validation study results. Mutat Res Genet Toxicol Environ Mutagen.

[26] OECD. Test No.489: In Vivo Mammalian Alkaline Comet Assay: OECD Guidelines for the Testing of Chemicals. Paris: OECD; 2016. 10.1787/9789264264885-en.

[27] OECD. Test No.475: Mammalian Bone Marrow Chromosomal Aberration Test: OECD Guidelines for the Testing of Chemicals. Paris: OECD; 2014. 10.1787/9789264224407-en.

[28] Andrew J (2009). Olaharski and Nina Gonzaludo, Identification of a Kinase Profile that Predicts Chromosome Damage Induced by Small Molecule Kinase Inhibitors. PLoS Comput Biol.

[29] Hayashi M (2016). The micronucleus test-most widely used in vivo genotoxicity test. Genes and Environment.

[30] Sato S-I, Tomita I (2001). Short-Term Screening Method for the Prediction of Carcinogenicity of Chemical Substances: Current Status and Problems of an in vivo Rodent Micronucleus Assay. J Health Sci.

[31] Beedanagari S, Chackalamannil S (2017). 4.11-Genetic Toxicology. Comprehensive Medicinal Chemistry III.

[32] Kirsch-Volders M (2003). Report from the in vitro micronucleus assay working group. Mutat Res.

[33] Tweats D.J (2007). Report of the IWGT working group on strategies and interpretation of regulatory in vivo tests: I. Increases in micronucleated bone marrow cells in rodents that do not indicate genotoxic hazards. Mutat Res Genet Toxicol Environ Mutagen.

[34] Ji P, Murata-Hori M, Lodish HF (2011). Formation of mammalian erythrocytes: Chromatin condensation and enucleation. Trends Cell Biol.

[35] Manwani D, Bieker JJ (2008). Chapter 2 The Erythroblastic Island. Current Topics in Developmental Biology.

[36] An X, Mohandas N (2011). Erythroblastic islands, terminal erythroid differentiation and reticulocyte maturation. Int J Hematol.

[37] Goel S (2018). CDK4/6 Inhibition in Cancer: Beyond Cell Cycle Arrest. Trends Cell Biol.

[38] Ki U (2010). Emergence of Micronuclei and Their Effects on the Fate of Cells under Replication Stress. PLoS ONE.

[39] Decordier I (2005). Influence of caspase activity on micronuclei detection: a possible role for caspase-3 in micronucleation. Mutagenesis.

[40] Murray AW (2004). Recycling the Cell Cycle: Cyclins Revisited. Cell.

[41] Jayapal SR (2016). Cyclin A2 regulates erythrocyte morphology and numbers. Cell Cycle.

[42] Sankaran VG (2012). Cyclin D3 coordinates the cell cycle during differentiation to regulate erythrocyte size and number. Genes Dev.

[43] Van Putten LM, Croon F (1958). The Life Span of Red Cells in the Rat and the Mouse as Determined by Labeling with DFP32 in Vivo. Blood.

[44] Belcher EH, Harriss EB (1959). Studies of red cell life span in the rat. J Physiol.

[45] Ludwig LS (2015). Genome-wide association study follow-up identifies cyclin A2 as a regulator of the transition through cytokinesis during terminal erythropoiesis. Am J Hematol.

[46] Chen K (2009). Resolving the distinct stages in erythroid differentiation based on dynamic changes in membrane protein expression during erythropoiesis. Proc Natl Acad Sci.

[47] Jayapal SR (2010). Down-regulation of Myc is essential for terminal erythroid maturation. J Biol Chem.

[48] Lee Y (2014). Cyclin D1-Cdk4 controls glucose metabolism independently of cell cycle progression. Nature.

